# Andrographolide demonstrates anti-proliferative activity in oral cancer by promoting apoptosis, the programmed cell death process

**DOI:** 10.22038/ijbms.2024.76691.16599

**Published:** 2024

**Authors:** Gauri Mansinh Kumbhar, Amol Dilip Jadhav, Supriya Kheur, Ladke Vaibhav Sunil

**Affiliations:** 1 Dr. D. Y. Patil Dental College & Hospital Dr. D. Y. Patil Vidyapeeth, Sant Tukaram Nagar, Pimpri, Pune. Maharashtra. India Pune: 411018; 2 Institute of Applied Biological Research and Development, a Division of Nirav BioSolutions Pvt Ltd, Aundh, Pune, India; 3 Dr. D. Y. Patil Madical College, Hospital & Research Centre. Dr. D. Y. Patil Vidyapeeth, (Deemed to be University), Sant Tukaram Nagar, Pimpri, Pune. Maharashtra. India Pune: 411018

**Keywords:** Andrographolide Apoptosis, Cell cycle, Gene expression, In-silico analysis, Mitochondrial membrane-, potential, Oral cancer

## Abstract

**Objective(s):**

Andrographolide has been studied on different types of human cancer cells, but very few studies have been conducted on oral cancer. The study aimed to evaluate the anticancer potential of Andrographolide on an oral cancer cell line (KB) through *in-silico* network analysis and *in vitro* assays.

**Materials and Methods:**

The *in-silico* analysis involved the determination of drug-likeness prediction, prediction of common targets between oral cancer and andrographolide, Protein-Protein Interactions (PPI), hub genes, top 10 associated pathways by Kyoto Encyclopaedia of Genes and Genomes (KEGG) pathway, gene ontology (GO), and molecular docking experiments. *In vitro* assays comprised MTT assay, apoptosis assay, cell cycle analysis, intracellular reactive oxygen species (ROS) measurement, mitochondrial membrane potential (MMP), anti-migration activity, and gene expressions using polymerase chain reaction (PCR).

**Results:**

Fifteen common genes were obtained and were seen to be involved in cellular proliferation, regulation of apoptosis, migration of cells, regulation of MAPK cascade, and regulation of cell cycle. The most common genes involved in the top 10 pathways were MAPK1, MAPK8, MAPK14, and IL6 which were seen to be associated with the MAPK signaling pathway which may be the key pathway through which andrographolide may aid in treating oral cancer. *In vitro* assays showed anti-proliferative properties, late apoptosis, and anti-migratory properties.

**Conclusion:**

According to the results obtained, andrographolide has shown anticancer properties and has the potential to be used as a chemotherapeutic drug. The *in-silico* approach used in the present study can aid as a model for future research in developing efficient cancer treatments.

## Introduction

Oral cancer (OC) has the unfortunate distinction of being the leading cancer in India, with an increasing incidence rate of 10.4% and a mortality rate of 9.3% for both men and women. On a global scale, it ranks as the eighth most prevalent cancer form (1). Despite the continued importance of surgical resection in cancer treatment, a major obstacle lies in the substantial risk of cancer recurrence observed in many patients, frequently occurring within a short period (2).

*Andrographis paniculata*, a herbal plant belonging to the *Acanthaceae* family, has been traditionally used in medicine for addressing a variety of health conditions. Widely cultivated in India, Thailand, and China (3), this plant’s leaves and stem are rich in the diterpene lactone known as andrographolide. *A. paniculata* showcases diverse bioactive properties, including anticancer effects (4), anti-inflammatory benefits (5), hepatoprotection (6), immunomodulation, and anticancer properties (7), as well as anti-infection properties (8). Through dichloromethane extraction, the primary bioactive compound andrographolide from *A. paniculata* has demonstrated the ability to hinder the proliferation of various human cancer cells, spanning a broad spectrum of cancer types (9). The primary bioactive compound found in the plant is andrographolide (C20H30O5), constituting 1.84% of the plant extract. This colorless, crystal-like compound possesses an intensely bitter taste and features a lactone structure, specifically a bicyclic diterpenoid lactone (10). Its molar mass is 350.455 g/mol and its melting point is 230-231 ^°^C. Its solubility in water is limited. In contemporary contexts, andrographolide and its various derivatives have been documented to exhibit numerous pharmacological attributes. These include anti-inflammatory, hepatoprotective, anti-viral, neuroprotective, antioxidant, anti-fibrotic, anti-hyperglycemic, anti-tumor, anti-atherosclerotic, antimicrobial, and cardiovascular protective properties (11-16).

Andrographolide has the capability to traverse the blood-brain barrier (BBB) and exerts a potent anti-inflammatory influence on various types of leukocytes, such as T-cells, macrophages, and neutrophils (17), as well as endothelial cells (18). Beyond its anticancer attributes, numerous studies have suggested that andrographolide exhibits a comprehensive anti-inflammatory impact, including the inhibition of NFκB binding to DNA (19, 20).

Andrographolide has been observed to regulate various signaling pathways associated with cancer and angiogenesis, including PI3K/AKT/mTOR (21, 22), SRC/MAPKs/AP-1 (23), TLR4/NF-κB/MMP-9 (24), and VEGF/VEGFR2/AKT (25). Recent findings underscore that the administration of andrographolide in human cancer cells elevates apoptosis rates and impedes cell proliferation. While this effect has been extensively studied in numerous cancer cells, the specific mechanistic pathways through which andrographolide exerts its anti-tumorigenic actions in OC remain unclear and await comprehensive understanding.

To explore the potential anti-cancer attributes of Andrographolide, particularly concerning OC, the present study utilized a combination of *in-silico* network pharmacology and *in vitro* assays.

## Materials and Methods

***In-silico analysis***

*Druglikeness prediction*

To assess the drug-likeness of andrographolide, Lipinski’s rule of five (RO5) was employed, for screening oral drugs in humans. Various parameters were evaluated. Andrographolide SMILES format, CC12CCC(C(C1CCC(=C)C2CC=C3C(COC3=O)O)(C)CO), was entered into the “SwissADME server (http://www.swissadme.ch)”, an online tool that calculates parameters like absorption, distribution, metabolism, excretion (ADME), oral bioavailability (OB), and drug-likeness (DL)(26). 

***Target proteins of andrographolide and oral cancer***

To predict the targets of andrographolide, the “Swiss Target Prediction database (http://www.swisstargetprediction.ch/)”(27) was employed for predicting the corresponding genes associated with andrographolide. The DisGeNet database was utilized to obtain the genes related to OC. The common genes were subsequently identified and selected for further analysis.

***Gene ontology and pathway enrichment***

To perform GO (Gene Ontology) and KEGG (Kyoto Encyclopedia of Genes and Genomes) pathway enrichment analysis, we utilized two different tools: the Database for Annotation, Visualization, and Integrated Discovery (DAVID, https://david.ncifcrf.gov/, ver. 6.8) for GO analysis and the ShinyGO database (ShinyGO, http://bioinformatics.sdstate.edu/) for pathway enrichment analysis. DAVID is a versatile tool for annotating and interpreting gene lists, while ShinyGO specializes in GO and pathway enrichment analysis. KEGG is a comprehensive pathway database that provides graphical representations of biochemical pathways(28, 29). GO is a valuable resource for functional genomics, offering definitions and classifications of gene functions (30). To present and analyze the data, we generated bubble charts and histograms using the Bioinformatics cloud platform (http://www.bioinformatics.com.cn/), an online platform designed for data processing and visualization.

***Protein-protein interaction analysis***

Protein-protein interactions play a crucial role in biological processes and are essential for understanding the complex systems within living cells (31). To map the PPI network, the cluster of target genes was analyzed using the Search Tool for the Retrieval of Interacting Genes database (http://string-db.org/; version 11.5). The analysis focused on “Homo sapiens” as the species and a threshold of >0.9 was applied to ensure high-confidence information. Subsequently, the PPI network was constructed using Cytoscape (https://cytoscape.org/; version 3.9.1), a widely used bioinformatics software for data visualization and integration (32). To identify clusters or highly interconnected regions within the PPI network, the Cytoscape plugin cytoHubba (https://apps.cytoscape.org/apps/cytohubba; version 0.1) was employed. Proteins with the highest MNC (Maximum Neighborhood Component) level rankings were identified as hub targets within the network.

***Molecular docking assessment between hub genes and andrographolide***

 The molecular docking simulations were executed using CB-Dock, a tool capable of automatically identifying active sites within a given protein, determining their centers and sizes, based on the query ligands (33). The Protein Data Bank (http://www.rcsb.org) was used to access the crystal structures of the target proteins. Similarly, the 3D structure of Andrographolide was obtained from the PubChem compound database (https://pubchem.ncbi.nlm.nih.gov/). These protein and ligand structures were used as inputs for CB-Dock, where the docking analysis explored the binding activities between the proteins and andrographolide. The Discovery Studio Visualizer software (Accelrys Software Inc.) was employed for the visualization and analysis of the docking results (34).

***Top of form***

*Gene expression levels of hub genes*

In this study, Gene Expression Profiling Interactive Analysis (GEPIA; http://gepia2.cancer-pku.cn/) was employed to verify the varied expressions of the hub genes in OC and normal oral tissues. GEPIA is an online server that offers interactive and customizable functionalities utilizing data from the Cancer Genome Atlas (TCGA) and Genotype-Tissue Expression (GTEx) database Furthermore, GEPIA facilitated the analysis of these genes based on pathological stages, providing valuable insights into their expression patterns in different disease stages (35).

*Overall survival analysis of hub genes*

To investigate the impact of the hub targets on the overall survival (OS) of patients with OC, the Kaplan-Meier [KM] Plotter (http://kmplot.com/analysis/index.php?p=service)(36), a cancer genomics dataset, was utilized. This dataset allows for the assessment of the prognostic significance of genes on survival outcomes. The patients with OC were categorized into two groups based on the presentation levels of the hub genes: low and high expression. A KM survival plot was generated to compare the survival outcomes between the two groups. 

***Anticancer activity of Andrographolide***

*Cell culture and maintenance*

The KB (Oral Cancer) cell line was obtained from the National Centre for Cell Sciences (NCCS) in Pune. These cells were cultured in DMEM (GibcoTM) medium at a temperature of 37 ^°^C. The culture medium was supplemented with 10% Fetal Bovine Serum (FBS, GibcoTM) and 1% antimycotic-antibiotic solution (GibcoTM). The cells were maintained in a CO_2_-enriched environment with a concentration of 5% to support their growth and viability.

***Preparation of stock solutions***

Andrographolide (Sigma-Aldrich) was prepared as a stock solution with a concentration of 10 mg/ml by dissolving 10 mg of andrographolide in 1 ml of Dimethyl Sulfoxide (DMSO). This stock solution was then stored at -80 ^°^C. From the stock solution, a working solution with a concentration of 1 mg/ml was prepared by diluting the stock solution in a complete medium and passing it through a sterile 0.22 µm filter to ensure sterility. Dilutions of the compound were made in a complete culture medium to obtain concentrations of 110, 100, 90, 80, 70, and 60 µg/ml. 

***Cytotoxicity assay of andrographolide***

The MTT conversion assay was employed to assess cell cytotoxicity. KB cells were seeded in a 96-well plate at a density of 5×10^4^ cells/ml. Various concentrations of andrographolide, ranging from 60 µg/ml to 110 µg/ml, were applied to the cells. After treatment, 20 µl of 5 mg/ml MTT solution was added to each well and incubated for 4 hr. Subsequently, 100 µl of DMSO was added to each well to dissolve the formazan crystals. The absorbance at 570 nm was measured using the Multi-scanGo Thermo Fischer Scientific ELISA plate reader. The IC50 values were calculated using a Microsoft Office Excel worksheet (37).

***Apoptosis analysis***

The apoptosis assay was performed using the FITC Annexin V/Dead Cell Apoptosis Kit (Invitrogen-Molecular Probes^®^). The protocol prescribed by the manufacturer was followed. Flow cytometry was used to examine these stained cells at emission wavelengths of 530 and >575 nm. In this assay, Andrographolide and Paclitaxel (PTX) IC50 and IC25 values were used for performing the Apoptosis assay (38)**.**

***Cell cycle analysis***

KB cells, both with and without treatment of andrographolide and PTX, were harvested 24 hr after washing with 1X PBS and trypsinization. To the collected cells, a mixture of 25 μl RNase A (20 mg/ml Invitrogen), 2 mM MgCl2 (Sigma), and 5-10 μl of 100 µg/ml propidium iodide (Invitrogen) was added. The cells were then incubated at room temperature for 10-15 min and subsequently analyzed using a FACS-caliber instrument from BD Bioscience (39). 

***Wound scratch assay***

 A density of 2×10^5^ cells/ml was seeded in 12-well plates and allowed to reach over 90% confluency. A linear wound was created in the center of each well using a 200 μl plastic tip. The wounded cell monolayers were washed three times with 1X PBS to remove cell debris and then treated with andrographolide and PTX, with a control group, before being incubated for 24 hr. Photos of the scratches were recorded at 0, 6, and 24 hr using an OLYMPUS CKX53 microscope. Subsequently, the cells were allowed to migrate by incubating them at 37 ^°^C in a medium containing 5% serum in the presence or absence of the drugs (40). 

***Estimation of generation of intracellular reactive oxygen species [ROS]***

To assess ROS generation, flow cytometry was employed using DCFHDA(41). In this assay, KB cells were cultured in 6-well plates for 24 hr. Upon reaching 70-80% confluence, the cells were treated with andrographolide and PTX at concentrations corresponding to their respective IC50 and IC25. Flow cytometry [Beckman Coulter Cytomics FC 500 instrument] (495 nm and 520 nm) was employed to measure the fluorescence. 

***Mitochondrial membrane potential (ΔΨm):***

To assess ΔΨm in KB cells, we employed the MitoProbe™ DiIC1 (5) Assay Kit as per the manufacturer’s instructions. This kit contains DiIC1 (5), a cyanine dye sensitive to changes in membrane potential, and CCCP, a disrupter of mitochondrial membrane potential used for research. DiIC1 (5) can readily enter cell cytoplasm and accumulate in mitochondria with active ΔΨm, yielding a bright far-red fluorescence. 

***RNA isolation and RT-PCR***

The culture cells were treated with the desirable drug concentrations. After drug incubation cells were harvested by discarding the growth medium and total RNA was isolated from cells using the TRIzol method (TRIzol™ Reagent, Invitrogen cat no. 15596018), following the manufacturer’s instructions. The primers used in the present study are listed in Supplementary Table 1[ST: 1].

***Statistical analysis***

The experiments were replicated three times, and the results are presented as the mean±standard deviation (SD). Data analysis was done using GraphPad Prism 8. “Two-way ANOVA” followed by respective *post hoc* tests at ****P<0.0001, ***P<0.001, and **P<0.01 to determine the statistical significance between the groups as compared to control.

**Table 1:** Molecular docking scores for andrographolide with hub target proteins depicting binding energy levels indicating strong interaction of hub gene and andrographolide

**Receptor**	**PDB ID**	**Binding energy (kcal/Mol)**
**IL6**	1P9M	-7.1
**MMP9**	1L6J	-7.2
**MAPK1**	1TVO	-9
**AR**	1XQ3	-7.5
**CDK4**	2W96	-8.1
**MAPK14**	1A9U	-7
**MAPK8**	3PZE	-7.8
**ADAM17**	2DDF	-7.7
**WEE1**	5VC5	-8.4
**CDK1**	4YC6	-7.9

**Figure 1:**
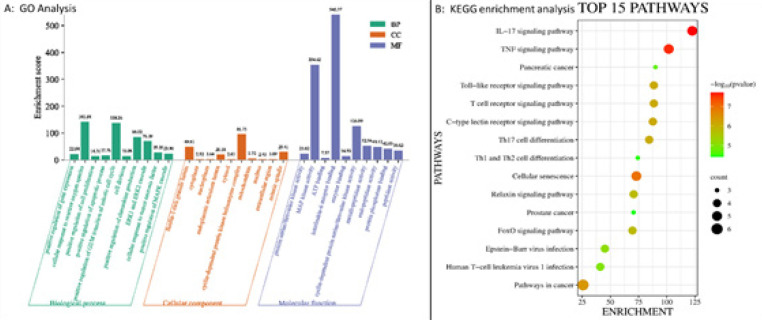
Gene ontology (GO) and kyoto encyclopaedia of genes and genomes (KEGG) enrichment analysis

A): GO enrichment analysis was obtained by taking 10 biological processes (BP), Molecular functions (MF), and Cellular components (CC) from the DAVID Database for the targets in andrographolide treating oral cancer. B): A Bubble plot depicting the enrichment of the top 15 signaling pathways associated with Oral Cancer was generated. The X-axis represents the enrichment factor of the genes, while the Y-axis represents the different pathways. The circles in the plot are color-coded and sized based on the Log10 [P-value], where red signifies pathways with the highest number of genes and light green indicates pathways with fewer genes

**Figure 2:**
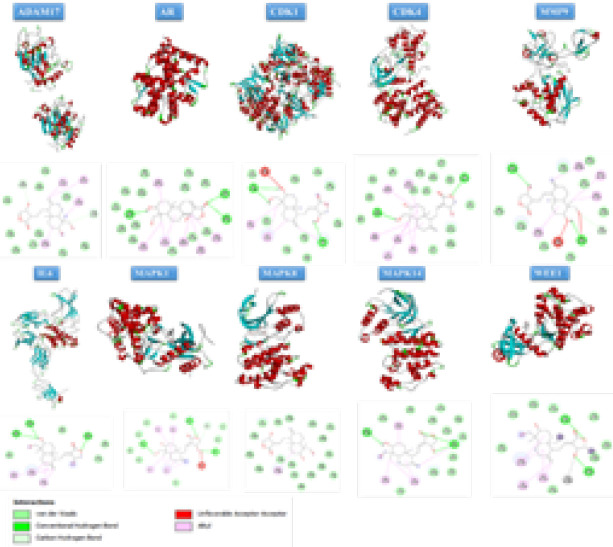
Sketch and matching diagrams of molecular docking of Andrographolide and top 10 hub genes (target proteins)

**Figure 3:**
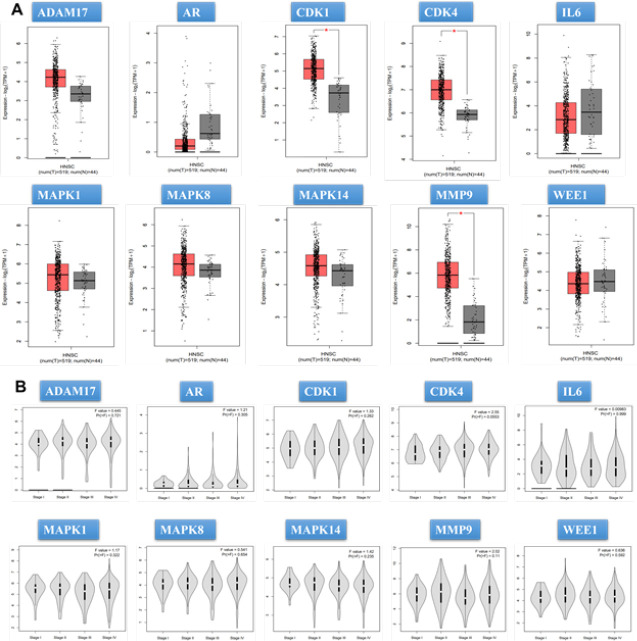
mRNA expression levels of hub genes in the cancer genome atlas (TCGA) and genotype-tissue expression (GTEx) databases

(A) mRNA expression levels in the GEPIA database (Boxplot of hub genes). Red depicts Oral Cancer tissue and grey depicts normal oral mucosa. (B) mRNA expression level and pathological stage in the GEPIA database. (Stage plot of hub genes)

**Figure 4:**
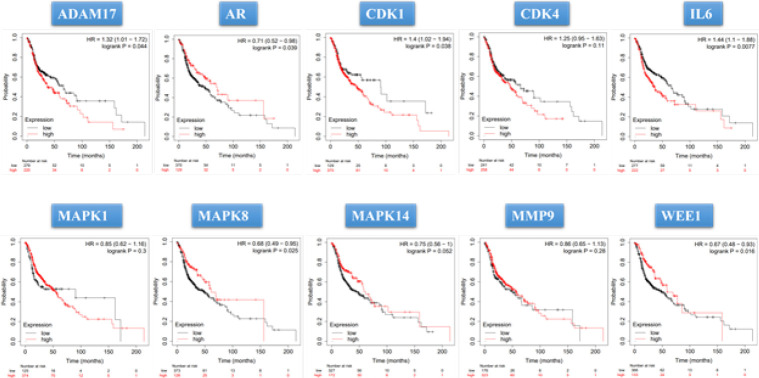
Kaplan–Meier overall survival analyses of patients with oral cancer based on expression of the ten hub genes. HR, hazard ratio (“http://kmplot.com/analysis/index.php?p=service&cancer=pancancer_rnaseq”)

**Figure 5:**
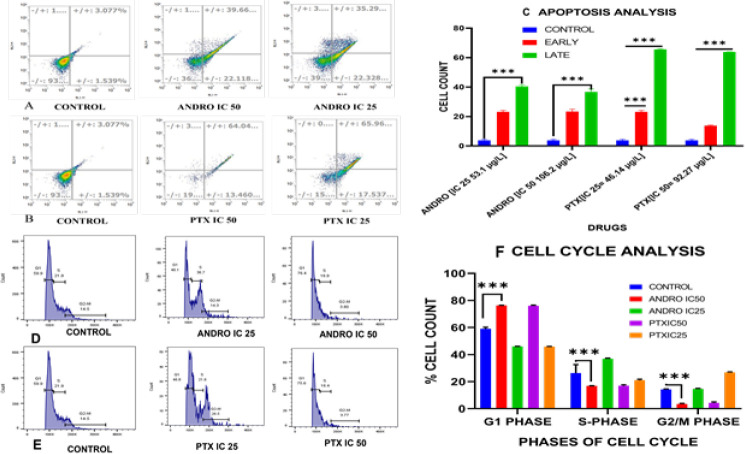
Apoptosis (A-C)

A) Flow-cytometry assay of andrographolide-induced apoptosis with andrographolide at IC_50_ (106.2 µg/ml) and IC_25_ (53.1µg/ml) after 24 hr, (B) represents the cells treated with PTX at IC_50_ (92.27 µg/l) and IC_25_ (46.13µg/ml) after 24 hr. (C) The data was expressed as mean±standard deviation (SD), comparing apoptotic phases for the respective concentrations of andrographolide and PTX. 5: Cell Cycle (D-F). D) Assessment of the impact of andrographolide and PTX on cell cycle distribution with concentration of andrographolide IC_50_ (106.2 µg/ml) and IC_25_ (53.1 µg/ml). E) PTX IC_50_ (92.27 µg/l) and IC_25_ (46.13µg/ml) for 24 hr. F) The data was expressed as mean±standard deviation (SD); comparing different phases of the cell cycle for the respective concentrations of andrographolide and PTX

**Figure 6:**
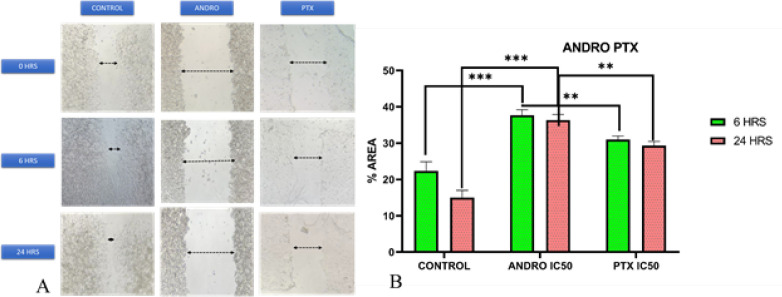
Representative Image for scratch assay

A) KB cells were incubated with andrographolide at IC50 (106.2 µg/ml) and PTX IC50 (92.21µg/ml) after 24 hr. The migration of KB cells was suppressed by andrographolide IC50 after 6 and 24 hr, as depicted in the second vertical panel. The first panel represented the control group. Additionally, the scratch area of KB cells treated with PTX IC50 was shown in the third panel. B) The data was expressed as Mean ± Standard Deviation (SD) of n=3 of independent experiments

**Figure 7:**
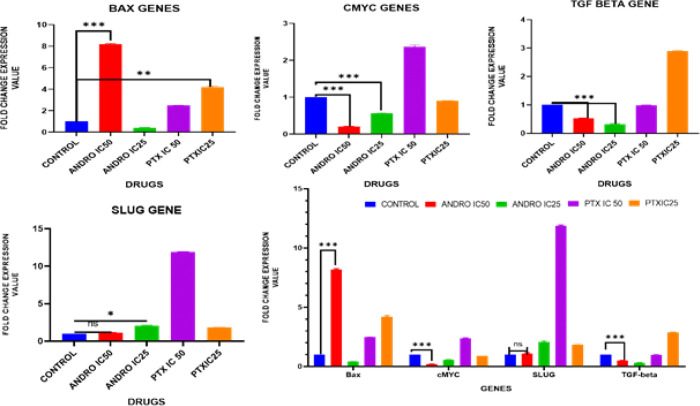
mRNA Expression levels of KB cells (Real time qRT-PCR)

Comprehensive analysis of Bax, c-Myc, SLUG, and TGF-β genes in KB cells treated with andrographolide and PTX at IC_50_ and IC_25_ with medium only. The b-actin gene is used as a control. The results were subjected to Two-way ANOVA followed by Tukey post hoc tests at ****P<0.0001, ***P<0.001, and **P<0.01 to determine the statistical significance between the groups as compared to the control of respective time points

## Results

***In-silico analysis***

*Molecular properties of andrographolide*

Our findings demonstrate that andrographolide adheres to Lipinski’s Rule of Five (RO5). The molecular properties of andrographolide, followed the RO5 criteria, indicating that it possesses favorable drug-like characteristics. 

***Target identification and analysis***

 Screening of OC-related targets using the search term “Lip and oral Cavity carcinoma” resulted in the identification of a total of 734 targets. Additionally, the Swiss Target Prediction database was employed to search for targets of andrographolide, leading to the identification of 100 targets. A comparison between the targets of andrographolide and the OC-related targets revealed a common set of 15 genes. (Supplementary Figure 1. SF:1). 

***Development of protein–protein interaction network (PPI) and determination of key targets***

The study used the STRING Database to analyze protein-protein interactions among 15 identified targets. Cluster analysis in Cytoscape 3.9.1 revealed two distinct clusters: Cluster 1 (*P*-value-0.00297)(SF:2) associated with cell proliferation (red), apoptosis (yellow), cell migration (blue), and the MAPK cascade (green), and Cluster 2 (*P*-value-0.00889)(SF:2) linked to cell division, G2/M transition regulation (red), and G1/S transition regulation in the mitotic cell cycle (blue). These clusters suggest close associations and shared biological processes among the target proteins.

***Top 10 hub genes analysis***

Through the application of various algorithms, the study identified the top 10 hub genes: IL6, MMP9, MAPK1, AR, CDK4, MAPK14, MAPK8, ADAM17, WEE1, and CDK1. Among these hub genes, IL6 emerged as the most prominently active gene (ST: 2, SF:3).

***GO and KEGG enrichment analysis***

GO enrichment analysis of the 10 hub genes revealed around 69 GO terms. In terms of biological processes (BP), these targets are involved in cellular responses to ROS, regulation of the G2/M phase, and apoptotic processes. The cellular component (CC) results included the cyclin-dependent protein kinase, mitotic spindles, and the endoplasmic reticulum lumen. In relation to molecular function (MF), the targets predominantly play roles in IL6 receptor binding and MAP kinase activity (Figure 1). Through KEGG pathway analysis, a total of 40 pathways were identified. Among these pathways, the top 15 pathways were selected for further examination. It was found that the top 10 hub genes had close associations with various pathways, predominantly the TNF signaling pathway, IL-17 signaling pathway, and cellular senescence (Figure 1). The top 10 pathways consistently involved genes such as MAPK 1, 8, 14, and IL-6. These genes were notably associated with the MAPK signaling pathway (ST:3). 

***Confirmation of hub target by molecular docking***

In a recent evaluation of drug-target interactions, ten hub genes were chosen as targets for molecular docking analysis. Using CB-DOCK, the structure of andrographolide was assessed for its docking potential with IL6, MMP9, MAPK1, AR, CDK4, MAPK14, MAPK8, ADAM17, WEE1, and CDK1. The results revealed binding energies lower than -5.0 for all core target proteins, indicating a robust binding activity between andrographolide and the core targets. The specific binding energies are presented in Table 1, and the docking sketch maps illustrating the interactions between the target proteins and andrographolide are depicted in Figure 2.

***mRNA expression levels of hub genes***

Using the GEPIA database, we found that CDK1, CDK4, and MMP9 mRNA levels were significantly higher in OC tissues compared to normal oral mucosa samples (*P*<0.01)(Figure 3A). Furthermore, results showed that CDK1, CDK4, IL6, MAPK8, and MMP9 exhibited significant changes across different pathological stages. CDK1, CDK4, IL6, and MAPK8 showed substantial increases in stage IV, while MMP9 increased in stage II (Figure 3B). 

***Survival analysis of the hub genes***

Survival analysis was conducted on the 10 hub genes. The analysis was performed on a cohort of 500 OC patients from the TCGA database. The results demonstrated that all of the hub genes exhibited a significant association with poor prognosis (*P*<0.05)(Figure 4).

***Anticancer activity of andrographolide on KB cell line:***

*Cytotoxicity result*

Using different concentrations of Andrographolide (60, 70, 80, 90, 100, and 110 µg/ml) and PTX (40, 50, 75, 100, 150, and 200 µg/ml) the experiment was performed and the findings demonstrate that andrographolide exhibited a potent cytotoxic effect on the KB cell line in a dose-dependent manner, with an IC50 value of 106±1 µg/ml and an IC25 value of 53±1 µg/ml. Similarly, PTX displayed an IC50 value of 92±4.43 µg/ml and an IC25 value of 46.1±2.21 µg/ml (SF:4).

***Effect of andrographolide on apoptosis regulation***

Apoptosis analysis of KB cells was conducted with two concentrations of andrographolide: IC50 and IC25 for 24 hr. Results showed that exposure to the IC50 concentration led to 22.22±0.1 cells in the early stage of apoptosis and 37.48±3.1 cells in the late stage of apoptosis. Similar observations were made with the IC25 concentration of Andrographolide (Figure 5A). Whereas PTX, at its IC50, resulted predominantly in late stage of apoptosis. Similar observations were made at the IC25 value of PTX (Figure 5B). Additionally, when comparing the effects of andrographolide and PTX, it was noted that both compounds had a more pronounced impact on late apoptosis compared to early apoptosis (Figure 5C).

***Effect of Andrographolide on cell cycle regulation of KB cells***

Andrographolide at concentrations corresponding to IC50 and IC25 for 24 hr showed that approximately 46.7±0.8% of cells were arrested in the G1 phase, with 36.15±0.9% in the S phase at IC25 value. Similarly, at the IC50 concentration, around 7642±1.0% of cells were arrested in the G1 phase, and 16.8±0.6% were in the S phase (Figure 5D, 5E). After 24 hr of treatment with PTX at the IC50 concentration, there was an accumulation of 75.85±0.21 cells in the G1 phase and 16.75±0.92 cells in the S phase. Similarly, at the IC25 concentration, 46.35±0.07 cells were observed in the G1 phase and 21.4±4.38 cells in the S phase (Figure 5F). This finding underscores the specific impact of andrographolide on halting cell cycle progression during the G1 phase, shedding light on its potential implications for therapeutic interventions.

***Effect of andrographolide on intracellular ROS measurement and mitochondrial membrane potential (ΔΨm) measurement***

While Andrographolide at IC25 showed a slight ROS decrease in cancer cells, it wasn’t statistically significant (SF:5). We used potentiometric dyes in flow cytometry to detect early apoptosis stages marked by ΔΨm loss, but Andrographolide at IC50 and IC25, along with PTX, did not affect mitochondrial membrane potential in KB cells (SF:6). Thus, these treatments did not impact the mitochondrial membrane potential of KB cells.

***Wound scratch assay results***

 After seeding the cells, a 24-hour incubation period was provided before treating them with IC50 concentrations of Andrographolide and PTX, alongside the control group. The wells were examined at 0, 6, and 24 hr to assess the effects of the treatments. The results indicated that the control group exhibited cell proliferation and migration after 6 hr, which was not observed in the group treated with Andrographolide and PTX (Figure 6A). The group treated with andrographolide and PTX showed significant inhibition of cell proliferation and migration at both 6 and 24 hr compared to the control group (Figure 6B).

***Andrographolide-induced apoptosis in KB cells by targeting genes***

Andrographolide caused an up-regulation of Bax gene expression predominantly by IC50 value, whereas IC25 did not show a significant effect on Bax expression. Whereas IC50 did not show any changes in SLUG expression. However, IC25 showed a slight increase in SLUG expression. indicating the induction of apoptosis in cancer cells and not affecting Epithelial-Mesenchymal Transition. Andrographolide induced apoptosis of OC cells by the TGF-β/c-myc pathway. At both IC-25 and IC-50 concentrations, andrographolide significantly reduced the expression of TGF-β and c-myc. IC50 value showed a significant reduction in c-myc expression compared to IC25 value. This observation suggests that andrographolide may inhibit c-myc expression by suppressing TGF-β, thereby regulating cellular proliferation and apoptosis (Figure 7).

Overall Andrographolide IC50 value exhibited a significant effect on the above-mentioned gene expression contributing to the anti-cancer activity of andrographolide against OC predominantly as an apoptosis promoter and regulation of cellular proliferation.

## Discussion

Despite ongoing research and treatment advancements, clinical outcomes and overall survival rates for HNSCC have seen limited improvement in recent decades, with a discouraging 5-year survival rate as low as 50% (42, 43). Given the unsatisfactory results and significant toxicity associated with current treatment approaches for HNSCC, current research is concentrated on exploring alternative therapies with reduced toxicity. Complementary and alternative medicine has gained increasing attention as a promising area for cancer management, leading to a greater focus on exploring these options in recent years. In the recent past, over 3000 anti-cancer products derived from plants have been introduced, and what’s intriguing is that they tend to have significantly fewer side effects compared to conventional chemotherapy drugs (44). 

An *in-silico* approach identified anti-cancer genes targeted by andrographolide. We assessed its cytotoxicity using the MTT assay and examined apoptotic potential through multiple assays in an OC cell line.

We identified 15 common genes associated with andrographolide and OC, impacting processes like cell proliferation, apoptosis regulation, cell migration, and the MAPK cascade. Notably, IL6, MMP9, MAPK1, MAPK14, and ADAM17 were prominent among the top 10 hub genes. Andrographolide demonstrated cytotoxic effects with an IC50 value of 106.2 µg/ml, inducing late apoptosis. This aligns with previous research where *A. paniculata* leaves’ methanol extract yielded four cytotoxic compounds, with andrographolide exhibiting the highest cytotoxicity and caspase-3 activation in HSC-2 cells (45). 

Andrographolide was found to induce apoptosis in HT-29 cells, linked to increased intracellular ROS levels and disruption of ΔΨm. Interestingly, it caused G2/M phase cell cycle arrest at lower doses and G0/G1 phase arrest at higher doses (46)**.** In contrast to some previous studies, we didn’t observe significant changes in ROS and MMP activity in our study. Andrographolide led to cell cycle arrest in the G0/G1 phase in our experiments.

Cancer cells often evade apoptosis by boosting anti-apoptotic BCL-2 proteins. Conversely, more resistant cancer types may down-regulate or inactivate pro-apoptotic proteins like Bax to suppress apoptosis. In our study, we noticed an up-regulation of Bax, suggesting that andrographolide exerts its anticancer effects by targeting the apoptotic pathway. Top of Form

Elevated c-Myc levels have been associated with reduced expression of immune checkpoints, which can suppress the immune response. In another study, inhibiting c-Myc expression was found to enhance apoptosis in CAL-27 cells (47). Another study by Marconi *et al*. emphasized the critical role of c-Myc in cell survival, proliferation, and tumor growth in Cal-27 cells (48). In accordance with these study results, our study also suggested that andrographolide may act as a negative regulator of c-Myc activity.

## Conclusion

Our network pharmacology analysis demonstrates its impact on various targets, pathways, and biological processes, effectively regulating cell proliferation and apoptosis to combat OC. This indicates it inhibits cell viability, induces apoptosis, and suppresses TGF-β and downstream gene c-myc, highlighting its anti-OC activity. By inhibiting c-myc, it stabilizes the cell cycle, proliferation, DNA synthesis, and genomic stability, potentially enhancing chemotherapy efficacy against OC. It is a promising candidate for tumor therapies and a chemopreventive agent in human OC treatment. Nonetheless, further research is needed to validate its clinical effectiveness and comprehensively understand its mechanisms in the context of OC.
